# Crystal Structure Stability of CL-20/MTNP Energetic Cocrystals Without/with Polydopamine Coating in a Water/Organic Solvent Environment

**DOI:** 10.3390/molecules31142527

**Published:** 2026-07-20

**Authors:** Peilin Yang, Yiru Chen, Chunbo Shi, Gang Li, Jinkun Guo, Kezhen Lv, Yeming Huang, Yijuan Liu, Yu Liu, Shiliang Huang, Xiaoan Wei

**Affiliations:** 1 School of Chemistry and Chemical Engineering, Nanjing University of Science and Technology, Nanjing 210094, China; yangpeilin23@gscaep.ac.cn (P.Y.); guojinkun22@gscaep.ac.cn (J.G.); 2 National Key Laboratory of Chemical Explosion Safety, Institute of Chemical Materials, China Academy of Engineering Physics, Mianyang 621999, China; chenyiru22@gscaep.ac.cn (Y.C.); shichunbo23@gscaep.ac.cn (C.S.); lkzh100@163.com (K.L.); huangyeming25@gscaep.ac.cn (Y.H.); liuyijuan25@gscaep.ac.cn (Y.L.); liuyu307@caep.cn (Y.L.)

**Keywords:** energetic cocrystal, crystal structure stability, chemical environment, decomposition, powder X-ray diffraction

## Abstract

Crystal structural stability is one of the core indicators for evaluating the safety of energetic materials, since these energetic materials undergo complex environments during manufacturing, processing, transportation, storage, and application in weapon systems. Energetic cocrystals are a novel solid form of energetic materials with unique crystal structures and novel properties. In this study, the crystal structure stability of hexanitrohexoazaisowurtzitane/1-methyl-3,4,5-trinitro-1H-pyrazole (CL-20/MTNP), an energetic cocrystal with high performance, in a water and organic solvent environment (acetone, *N*,*N*-dimethylacetamide, dimethyl sulfoxide and ethanol) is characterized. It was found that CL-20/MTNP partially decomposed and transformed to *α*-CL-20 both in pure water and a mixture of water and organic solvent, as confirmed by powder X-ray diffraction. The decomposition temperature of CL-20/MTNP and residual solid phase in a chemical environment were observed to be significantly different under thermal stimulation. The degree of the decomposition can be controlled by the temperature and soaking time. Unexpectedly, no significant changes were observed for the decomposition behavior after the introduction of organic solvents. Moreover, a polydopamine coating method was also applied to inhibit the decomposition of the cocrystal structure of CL-20/MTNP. These results are of significant reference value for understanding the crystal structure stability of energetic cocrystals and evaluating their safety in practical applications.

## 1. Introduction

Crystal structural stability is one of the core indicators for evaluating the safety of energetic materials, since these energetic materials undergo complex environments during manufacturing, processing, transportation, storage, and application in weapon systems [1,2,3,4,5]. Energetic cocrystals are a novel solid form of energetic materials, which possess unique crystal structures and synergistic complementary effects from multiple energetic molecules, enabling them to overcome the limitations of traditional single-molecule-component explosives and endowing energetic materials with novel properties. In the cocrystal structure, different energetic molecules are primarily bound together through non-covalent intermolecular interactions such as hydrogen bonding and π-π stacking [6,7,8,9,10], which are relatively weaker than covalent bonds. Therefore, the crystal structure stability of the crystal structure is of particular concern in the application of energetic cocrystals. Because of compositional heterogeneity and surface effect, various energetic cocrystals have been observed to exhibit significantly different thermal decomposition behaviors under thermal stimulation compared to single-molecule-component explosives [11,12]. Hexanitrohexoazaisowurtzitane/1-methyl-3,4,5-trinitro-1H-pyrazole (CL-20/MTNP) is an energetic cocrystal which exhibits an excellent comprehensive performance with reduced sensitivity (IS = 6 J, FS = 180 J), high density (1.932 g/cm^3^ at 293 K) and superior detonation performance (D = 9.35 km/s, P = 40.5 GPa) [13]. In our previous work [14], an unexpected low decomposition temperature was observed for CL-20/MTNP under isothermal conditions, revealing a surface-induced decomposition mechanism based on the characterization results of high-speed microscopy (HSM), scanning electron microscopy (SEM), in-situ powder X-ray diffraction (PXRD), and thermogravimetry-differential scanning calorimetry (TG-DSC). Besides thermal stimulation, energetic materials are also exposed to complex chemical environments in practical applications. For instance, organic solvents are normally used for the recrystallization of energetic materials to achieve crystals with high quality [15,16], and a certain humidity is critical to the storage safety of energetic materials. In addition, energetic materials are usually coated with polymer binders in water and organic solvents before they are compressed into polymer-bonded explosive (PBX) products [17,18,19]. Therefore, understanding the crystal structure stability of energetic materials under chemical stimulation is of great value for ensuring the safety of energetic cocrystals during manufacturing and storage processes. Here, the crystal structure stability of CL-20/MTNP in water and organic solvent environments (acetone, *N*,*N*-dimethylacetamide, dimethyl sulfoxide and ethanol) were characterized by the quantitative analysis of phase composition variation based on PXRD. Moreover, a polydopamine (PDA) coating method was also applied to inhibit the decomposition of the cocrystal structure of CL-20/MTNP [20,21,22,23]. These results are of significant reference value for understanding the crystal structure stability of energetic cocrystals and evaluating their safety in practical applications.

## 2. Experimental

### 2.1. Materials

Raw CL-20/MTNP cocrystal and pure water were provided by the Institute of Chemical Materials, China Academy of Engineering Physics. The acetone (AC), *N*,*N*-dimethylacetamide (DMF), dimethyl sulfoxide (DMSO), and ethyl alcohol (EtOH) were purchased from Chengdu Kelong chemical reagent factory. All the solvents were of analytical grade and used as received, without further purification.

### 2.2. Purification of Raw CL-20/MTNP Cocrystal

The raw CL-20/MTNP cocrystal was impure. To ensure the accuracy of the subsequent determination of the decomposition behavior of the CL-20/MTNP cocrystal, recrystallization was necessary. Raw CL-20/MTNP cocrystal (30 g) was added into 300 mL of 1:1 (*v*/*v*) acetone/ethanol solvent and completely dissolved through mechanical stirring. After filtration, the filtrate was stirred with a stirring speed of 250 rpm, until the liquid became turbid (i.e., crystals started to precipitate). Then, the stirring speed of the paddle was reduced to 100 rpm. When the mixed solution evaporated to approximately one-third of its original volume, stirring was stopped. The cocrystals were collected by filtration using a funnel and medium-speed filter paper, and then washed three times with pure water to remove impurities. Finally, the cocrystal was transferred to a vacuum oven and dried at 50 °C to obtain the pure CL-20/MTNP cocrystal. PXRD data collection and refinement were conducted for the raw CL-20/MTNP cocrystal and the purified CL-20/MTNP cocrystal after recrystallization, which confirmed that *α*-CL-20 was doped in the raw CL-20/MTNP cocrystal, and the CL-20/MTNP cocrystal was purified via the recrystallization method, as shown in Figure S1.

### 2.3. Decomposition of CL-20/MTNP Cocrystal in Pure Water

The CL-20/MTNP cocrystal was heated and soaked in pure water, and 100 mg of CL-20/MTNP cocrystal was added into 200 mL pure water. A glass rod was utilized to gently touch the cocrystal to make sure the crystals were evenly distributed at the bottom of the beaker. Then, the beaker was sealed with aluminum foil paper to reduce the evaporation rate of pure water. The beaker was placed in the silicone oil and heated while stirring at different temperature points (40 °C, 50 °C, 60 °C, and 70 °C) and different soaking durations (8 h, 16 h, 24 h, and 48 h). Three sets of parallel experiments were arranged simultaneously. The treated solid mixture was obtained by filtering with quantitative filter paper.

### 2.4. Decomposition of CL-20/MTNP Cocrystal in Water/Solvent Mixture

In the mixed solvent environment, the process was the same as in the pure water environment. Pure water was replaced by 200 mL of 10 mmol/L mixed water solution. CL-20/MTNP cocrystal (100 mg) was added to pure water, using a glass rod to make the crystals sink. Then, the solvents of AC, DMF, DMSO, and EtOH were added into the mixed solution, respectively. After that, the beaker was sealed with aluminum foil and heated in an oil bath at the same temperature used for the pure water system. The samples were heated from room temperature to the target temperatures and maintained at constant temperatures for 24 h.

### 2.5. Polydopamine-Coated CL-20/MTNP Cocrystal

The Tris (0.05 mol) was added into a beaker containing 450 mL pure water, and the mixture was vortexed until complete dissolution and homogeneous dispersion. A total of 5.1 mL of dilute hydrochloric acid was dropwise added under continuous vortexing to adjust the pH to 9.51. Then, the Tris buffer solution (0.1 mol/L, pH = 9.51) was obtained.

Two polydopamine coating strategies were adopted to fabricate PDA-modified CL-20/MTNP cocrystals. A total of 200 mL as-prepared Tris buffer was transferred into a reagent bottle and dopamine hydrochloride (8 mmol) was added and mixed to produce a 40 mM dopamine buffer solution. Subsequently, 1 g pure CL-20/MTNP cocrystal was immersed into the above buffer and vibrated to ensure the cocrystals were sunk below the liquid surface. Afterwards, sodium periodate (16 mmol) was added and dissolved. The products were collected via filtration, washed three times with pure water, and named CL-20/MTNP@PDA1.

The second coating route was performed in the same alkaline Tris medium without sodium periodate. Then, 1 g of CL-20/MTNP cocrystal was added into 100 mL 40 mM dopamine buffer and vibrated for complete submersion. The suspensions were continuously shaken for 10 min, followed by static settling for 1 min, 10 min, and 20 min, respectively. After they had been washed three times using pure water and filtering, the products were obtained and named CL-20/MTNP@PDA2, CL-20/MTNP@PDA2-10min, and CL-20/MTNP@PDA2-20min, respectively.

### 2.6. Characterization

SEM (Ultra 55, Carl Zeiss, Oberkochen, Germany) operating at an electron-gun accelerating voltage of 2 kV was utilized to examine the specimen morphology. Fourier transform infrared (FTIR) measurements were performed on a Thermo Scientific Nicolet 6700 infrared spectrometer (Nicolet, Glendale, WI, USA). TG-DSC instruments (Mettler-Toledo Co., Ltd., Greifensee, Switzerland) were employed for thermal stability assessment of samples, where nitrogen served as the protective atmosphere and the heating speed was controlled at 10 °C/min. The crystal phase identification and quantitative analysis of the solid product after soaking in the solvent were carried out using the method of PXRD refinement. The PXRD data were obtained on a D8 Advance X-ray diffractometer (Bruker, Karlsruhe, Germany) with Cu *K*_α_ radiation (*λ* = 0.154060 nm), accelerating voltage of 40 kV, and current of 40 mA. To improve the accuracy of quantitative analysis of crystal phases, when collecting the PXRD data of the cocrystal decomposition products, the step size was set to 0.01°, and the exposure time was set to 0.4 s per step.

### 2.7. Phase Analysis

PXRD data were processed via Jana2020 software for phase identification and quantitative Rietveld refinement.

First, experimental diffraction patterns and CIF crystal models of CL-20/MTNP and potential impurity phases were imported for Le Bail fitting. Zero shift, unit cell parameters, peak shape, and background parameters were refined without fixing atomic coordinates to complete qualitative phase screening.

After full convergence of structural parameters, Rietveld refinement was performed on the converged dataset to calculate the volume fraction of each crystalline phase. Atomic positions were fixed throughout quantitative fitting, and the overall polarity factor (*T*_Overall_) was optimized iteratively to match calculated and measured diffraction intensities, yielding reliable phase volume percentages of solid residues. Te dhetailed software workflow is provided in Supporting Information.

## 3. Results and Discussion

### 3.1. The Structural Stability of the CL-20/MTNP Cocrystal in Pure Water

**Figure 1:**
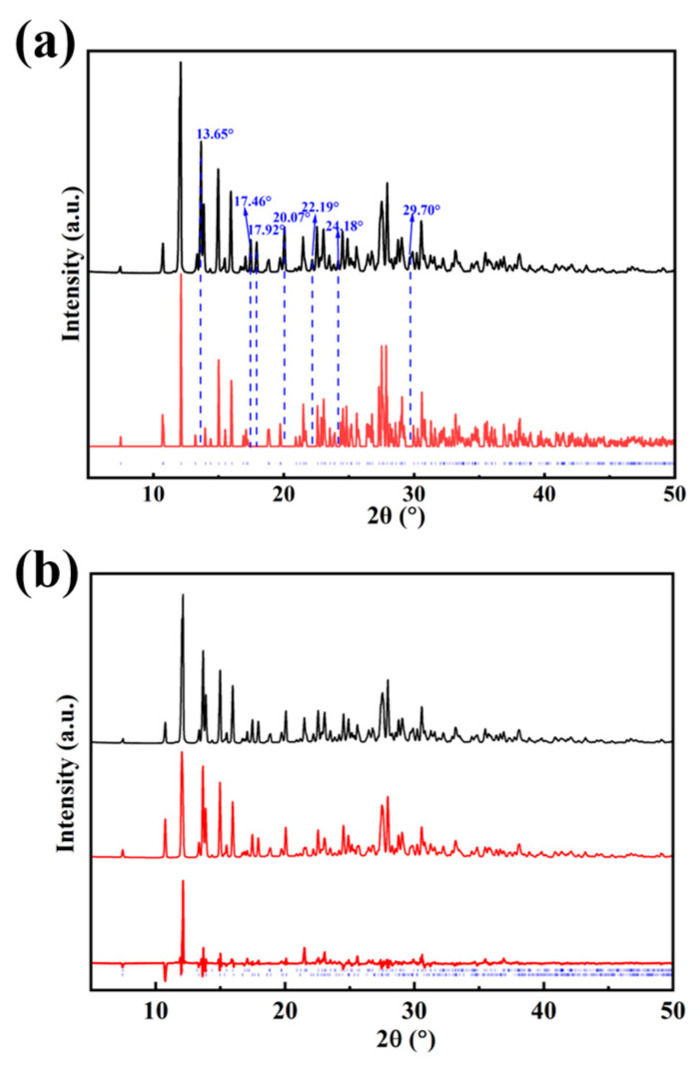
(**a**) Experimental PXRD pattern (black) of solid residues obtained from CL-20/MTNP cocrystal immersed in pure water at 70 °C for 48 h, alongside the simulated PXRD pattern (red) of CL-20/MTNP cocrystal. Vertical blue bars denote calculated peak positions; (**b**) Rietveld plot for the aforementioned solid residue after two-phase refinement (*Rp* = 0.0497, *φ*_a-CL-20_ = 0.505) showing experimental (black), calculated (red), and difference (red, bottom) patterns. Vertical blue bars denote calculated peak positions for CL-20/MTNP (1st raw) and a-CL-20 (2nd raw).

The experimental PXRD patterns of the solid residues after the treatment of pure CL-20/MTNP cocrystal at 70 °C for 48 h in a pure water environment are compared with the simulated PXRD pattern of pure CL-20/MTNP, as shown in Figure 1a. It can be observed that some diffraction peaks at 13.65°, 17.46°, 17.92°, 20.07°, 22.19°, 24.18°, and 29.70° are clearly unidentifiable, indicating that the CL-20/MTNP cocrystal undergoes decomposition to some extent. To identify the crystalline phases of the decomposition products, the CIFs of *α*-CL-20, *γ*-CL-20, and *ε*-CL-20 phases were imported for Rietveld refinement, as shown in Figure 1b and Figure S2a,b and the crystallographic data and characteristic peaks of them are listed in Table S1. All peaks of *α*-CL-20 coincide with the newly emerging peaks, indicating that the decomposition products of the CL-20/MTNP cocrystal in pure water are *α*-CL-20.

**Figure 2:**
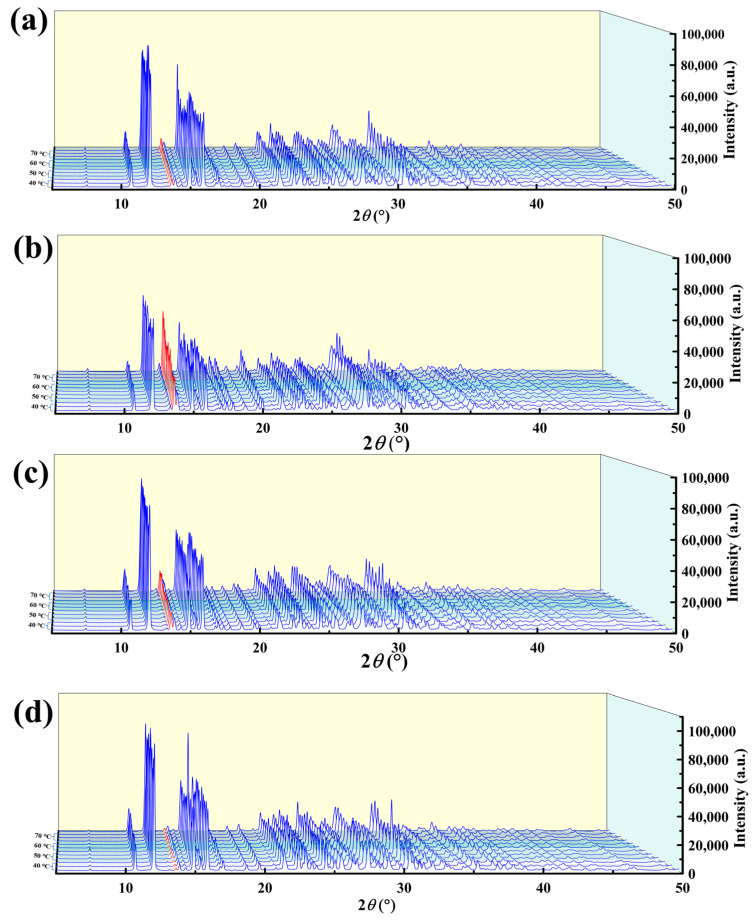
PXRD patterns of the solid residues obtained by treating CL-20/MTNP cocrystal in pure water at four temperatures for durations of 8, 16, 24, and 48 h: (**a**) 40 °C, (**b**) 50 °C, (**c**) 60 °C, (**d**) 70 °C. The *z*-axis represents immersion durations with three parallel samples. Patterns from parallel samples with identical soaking duration share the same background color. The characteristic peak of *α*-CL-20 marked in red.

The PXRD images are grouped by temperature and then compiled into 3D waterfall plots, as shown in Figure 2a–d. It can be observed that the 13.6° characteristic peak (marked in red) of *α*-CL-20 gradually intensifies with increasing immersion duration of the CL-20/MTNP cocrystal in water at each temperature. Namely, at any temperature, the longer CL-20/MTNP cocrystal is immersed in water, the higher the decomposition degree.

Following refinement and quantitative analysis of the PXRD data, the volume fraction of the secondary phase *ϕ_α_*_-CL-20_ is obtained, as shown in Table S2. It is worth noting that when the volume fraction of *α*-CL-20 is no more than 50%, the secondary phase corresponds to *α*-CL-20. However, when this value exceeds 50%, *α*-CL-20 is defined as the primary phase, and the volume fraction of the secondary phase equals that of the residual CL-20/MTNP cocrystal (*ϕ*_CM residue_). On this occasion, the volume fraction of α-CL-20 is *ϕ*_CM residue_. Based on formulas 1 to 3, the molar ratio of α-CL-20 (*χ_α_*_-CL-20_) are calculated and listed in Table 1, representing the initial decomposition molar ratio of CL-20/MTNP.

*V_α_*_-CL-20_/*V*_CM residue_ = *ϕ_α_*_-CL-20_/(1 − *ϕ_α_*_-CL-20_) (1)

n*_α_*_-CL-20_/n_CM residue_ = (*V_α_*_-CL-20_/*V*_CM residue_) × (*ρ_α_*_-CL-20_/*ρ*_CM_) × (M_CM_/M*_α_*_-CL-20_) (2)

*χ_α_*_-CL-20_ = n*_α_*_-CL-20_/(n*_α_*_-CL-20_ + n_CM residue_) = 1 − 1/(n*_α_*_-CL-20_/n_CM residue_ + 1) = 1 − 1/[(*V_α_*_-CL-20_/*V*_CM residue_) × (*ρ_α_*_-CL-20_/*ρ*_CM_) × (M_CM_/M*_α_*_-CL-20_) + 1] (3)

where *V* is volume, n is molar quantity, M is molecular weight, and *ρ* is density. The detailed refinement patterns after quantitative analysis are presented in Figures S3–S6, and the detailed calculation results of sample 1#–3# are listed in Table S3.

**Figure 3:**
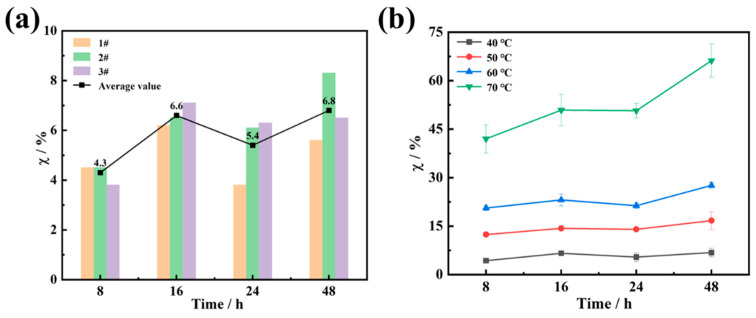
(**a**) The mole fraction of *α*-CL-20 from the decomposition products of CL-20/MTNP cocrystal immersed in pure water at 40 °C; (**b**) The variation of mole fraction of *α*-CL-20 from CL-20/MTNP cocrystal soaked in pure water at different temperatures for soaking times of 8–48 h.

At the same temperature, the trend of the mole fraction of *α*-CL-20 in the decomposition products with different soaked durations is shown in Figure 3 and Figure S7a–d. From 40 °C to 70 °C, the content of *α*-CL-20 in the products increases, with *χ_α_*_-CL-20_ rising from 4.3% to 6.8% at 40 °C, from 12.4% to 16.7% at 50 °C, from 20.6% to 27.6% at 60 °C, and from 42.0% to 66.2% at 70 °C, which suggests that temperature can promote the decomposition behavior of CL-20/MTNP cocrystal in a pure water environment. As shown in Figure 3b, from 60 °C to 70 °C, the *χ_α_*_-CL-20_ sharply increases by 38.6% after immersion in pure water for 48 h, which might be related to the energy barrier for the dissolution of MTNP molecules from the surface of the CL-20/MTNP cocrystal.

### 3.2. The Structural Stability of the CL-20/MTNP Cocrystal in Mixed Solution

**Figure 4:**
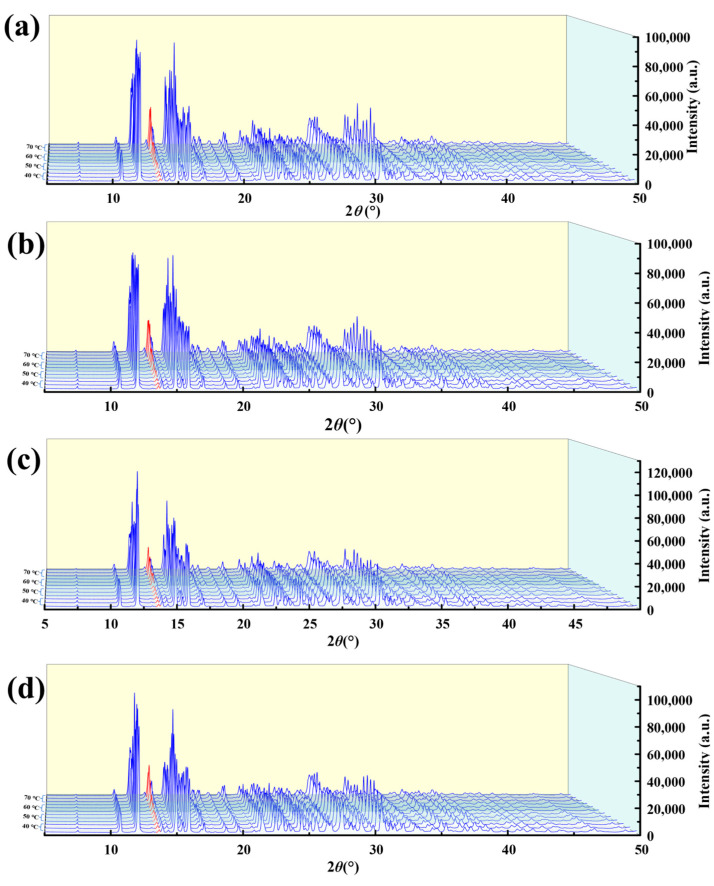
The 3D waterfall PXRD patterns for CL-20/MTNP cocrystal soaked for 24 h in four different aqueous mixed solvents at 40, 50, 60, and 70 °C: (**a**) 10 mmol/L AC aqueous solution, (**b**) 10 mmol/L DMF aqueous solution, (**c**) 10 mmol/L DMSO aqueous solution, (**d**) 10 mmol/L EtOH aqueous solution. The *z*-axis represents four temperatures with three parallel samples. Patterns from parallel samples with identical temperature share the same background color. The characteristic peak of *α*-CL-20 marked in red.

The CL-20/MTNP cocrystals are soaked in the various mixed solutions for 24 h. Decomposition products of CL-20/MTNP cocrystal in the mixed solutions, including AC aqueous solution, DMF aqueous solution, DMSO aqueous solution, and EtOH aqueous solution, are subjected to phase determination and quantitative analysis, as shown in Figures S8–S11, respectively. The volume fractions of *α*-CL-20 in these mixed solution systems are listed in Table S4. The results show that in these mixed solvents, the decomposition products of the CL-20/MTNP cocrystal are still *α*-CL-20, indicating that the addition of organic solvents does not affect the crystal phase of the CL-20 component in the CL-20/MTNP cocrystal decomposition products. As shown in Figure 4, for samples immersed for 24 h in the four mixed solvents, the characteristic diffraction peak at 13.6° assigned to α-CL-20 gradually intensifies upon rising temperature, which corresponds to a general increase in α-CL-20 content.

Based on Formula (3), the molar ratio of CL-20/MTNP cocrystal in the mixed solution is calculated after 24 h of immersion at 40, 50, 60, and 70 °C and summarized in Table 2 to quantitatively evaluate the decomposition behavior of the cocrystal in various solvents. The detailed information of *χ_α-CL-20_* of sample 1#–3# in a mixed solution environment is listed in Table S5.

**Figure 5:**
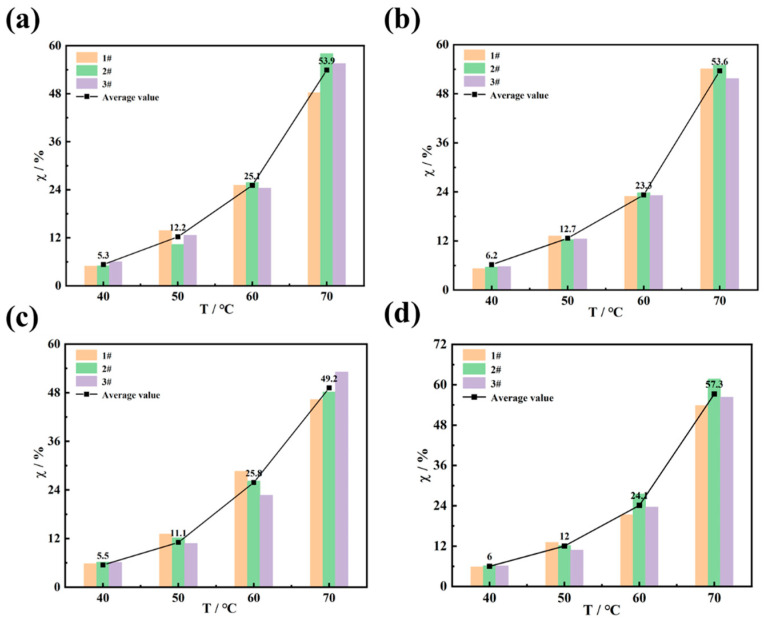
The mole fraction of *α*-CL-20 from CL-20/MTNP cocrystal soaked for 24 h at 40, 50, 60, and 70 °C in 10 mmol/L aqueous mixed solution: (**a**) AC, (**b**) DMF, (**c**) DMSO, (**d**) EtOH.

As shown in Figure 5a,b, the decomposition rates of the cocrystal in 10 mmol/L AC aqueous solution are 5.3% at 40 °C, 12.2% at 50 °C, 25.1% at 60 °C, and 53.9% at 70 °C, and in 10 mmol/L DMF aqueous solution, they are 6.2% at 40 °C, 12.7% at 50 °C, 23.3% at 60 °C, and 53.6% at 70 °C. There is barely any change at 40 °C and 50 °C, but a slight increase occurs at 60 °C and 70 °C by comparison with the results under pure water conditions (5.4% at 40 °C, 14% at 50 °C, 21.3% at 60 °C, and 50.7% at 70 °C). Moreover, as exhibited in Figure 5c, in the cocrystal in 10 mmol/L DMSO aqueous solution, the decomposition rates of the cocrystal are 5.5% at 40 °C, 11.1% at 50 °C, 25.8% at 60 °C, and 49.2% at 70 °C, respectively. Compared with the results under pure water conditions, there is a clear decrease in both the low-temperature zone and the high-temperature zone. As shown in Figure 5d, when the CL-20/MTNP cocrystal is in 10 mmol/L EtOH aqueous solution, the decomposition rates of the cocrystal are 6.0%, 12.0%, 24.1%, and 57.3%, corresponding to 40 °C, 50 °C, 60 °C, and 70 °C, respectively. Compared with the results under pure water conditions, there is no significant change in the low-temperature region, but there is a clear increase in the high-temperature region. The more detailed information for the comparison chart showing the variation in the mole fraction of *α*-CL-20 in different mixed solutions with temperature is exhibited in Figure S12.

### 3.3. Decomposition Inhibition by PDA Coating

In our previous work [14], it was found that the CL-20/MTNP cocrystal has a unique channel-like structure, which reduces the decomposition energy barrier of the CL-20/MTNP cocrystal and causes MTNP to escape along the channels under external stimulation. Based on this, we speculate that coating the surface of the cocrystal may effectively inhibit the escape behavior of MTNP. As a commonly used coating material in the field of energetic materials, dopamine can form a PDA layer through polymerization, which can fill the defects on the surface of energetic materials and reduce the generation of hot spots, providing a feasible material choice and theoretical basis for the coating modification of CL-20/MTNP cocrystal.

**Figure 6:**
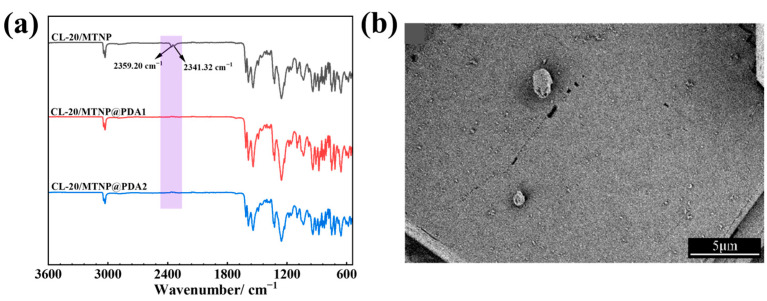
(**a**) Infrared spectra of CL-20/MTNP@PDA1 and CL-20/MTNP@PDA2-10min; (**b**) SEM images of CL-20/MTNP@PDA2-10min.

PXRD data collection and refinement analysis are performed on CL-20/MTNP cocrystal before and after PDA coating. The results show that after coating PDA in an environment of pH = 9.5, no other unspecified diffraction peaks appear in the PXRD patterns, indicating that the CL-20/MTNP cocrystal does not undergo structural decomposition during the coating process and maintains a stable cocrystal state, as shown in Figure S13. It should be noted that PDA has no obvious diffraction peaks, and the success of PDA coating therefore cannot be confirmed by PXRD diffraction results. As shown in Figure 6a, compared with CL-20/MTNP cocrystal, the infrared spectra of CL-20/MTNP@PDA1 and CL-20/MTNP@PDA2-10min lack the characteristic peaks at 2359.20 cm^−1^ and 2341.32 cm^−1^, which confirms that PDA is successfully coated on the surface of CL-20/MTNP cocrystal.

To clarify the coating effect of PDA on CL-20/MTNP cocrystal, SEM tests are carried out on CL-20/MTNP cocrystal, CL-20/MTNP@PDA1, and CL-20/MTNP@PDA2-10min samples. As shown in Figure S14a,b, compared with the smooth and flat surface of CL-20/MTNP cocrystal raw material, a large number of small particles appear on the surface of CL-20/MTNP@PDA1, which are speculated to be PDA particles formed by self-polymerization after one layer of PDA coating. The reason for the rough surface of CL-20/MTNP@PDA1 is presumably related to the preparation method. During its preparation, the coating rate of PDA is relatively fast, leading to the self-polymerization of PDA, while it is simultaneously coated on the surface of CL-20/MTNP cocrystal. Meanwhile, the self-polymerized small particles that form after one layer of coating are further coated on the cocrystal surface. In addition, this preparation method produces a large number of self-polymerized PDA particles. When sodium periodate (NaIO_4_) is added, the solution changes from light brown to black. During the filtration process, the viscous black mud-like self-polymerized PDA particles are difficult to separate from the CL-20/MTNP@PDA product, which further results in a significantly low yield of CL-20/MTNP@PDA. By maintaining an alkaline environment of pH = 9.5, without the addition of NaIO_4_, and extending the coating time, CL-20/MTNP@PDA2-10min and CL-20/MTNP@PDA2-20min samples were prepared. As shown in Figure 6b and Figure S14c,d, the surface of CL-20/MTNP@PDA2-10min is significantly smoother than that of CL-20/MTNP@PDA1 and CL-20/MTNP@PDA2-20min. Therefore, the samples of CL-20/MTNP@PDA2-10min were selected for further analysis of thermal and chemical stability.

**Figure 7:**
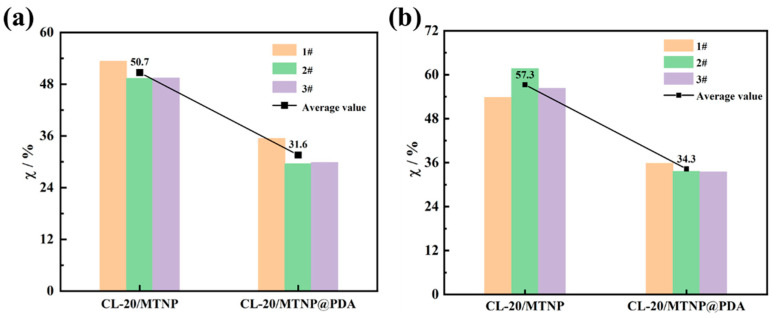
The mole fraction of decomposed CL-20/MTNP and CL-20/MTNP@PDA after 24 h immersion at 70 °C: (**a**) pure water; (**b**) 10 mmol/L aqueous EtOH solution.

The decomposition behavior of CL-20/MTNP@PDA2-10min in pure water and mixed solution systems is tested. As shown in Figure 7a,b, the decomposition molar ratio of CL-20/MTNP@PDA2-10min in a solution environments is compared with that of CL-20/MTNP cocrystal. The results show that the structural decomposition rate of CL-20/MTNP cocrystal in pure water decreased from 50.7% to 31.6%, and the structural decomposition rate of CL-20/MTNP cocrystal in 10 mmol/L ethanol aqueous solution decreased from 57.3% to 34.3%. These results indicate that PDA coating can, to some extent, reduce the decomposition degree of CL-20/MTNP cocrystal in a solution environment, but it cannot completely prevent the decomposition of the cocrystal. The reason is that the PDA coating on the surface can block the pores through which MTNP escapes from the cocrystal structure. However, since the surface PDA coating layer is not dense, especially at the edges and corners of the crystal, although a large portion of the MTNP molecules are blocked, a small amount of MTNP molecules still escapes, thereby causing local decomposition of the cocrystal.

### 3.4. Thermal Stability After PDA Coating

**Figure 8:**
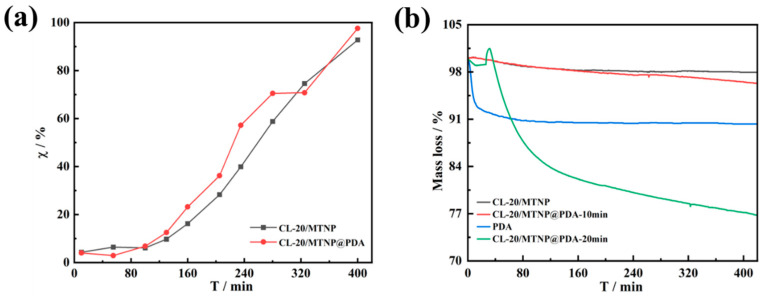
(**a**) Variation in mole fraction of *γ*-CL-20 from CL-20/MTNP and CL-20/MTNP@PDA2-10min with isothermal holding for 10 to 400 min at 180 °C during in-situ XRD test; (**b**) The weight loss curves of CL-20/MTNP, pure PDA, CL-20/MTNP@PDA2-10min, and CL-20/MTNP@PDA2-20min at an isothermal condition of 140 °C.

By conducting a 10 to 400 min holding at 180 °C, the in-situ PXRD analysis method is employed to study the stability of the CL-20/MTNP cocrystal and CL-20/MTNP@PDA under thermal stimulation. As shown Figure S15a,b, the characteristic peaks at 12.5°, 12.9°, and 17.5° are assigned to the decomposition product (*γ*-CL-20), and the intensity of these peaks gradually increased with the extension of holding time, while the intensity of the characteristic peak of CL-20/MTNP cocrystal at 12.1° gradually decreased. The decomposition product molar ratios of CL-20/MTNP cocrystal and CL-20/MTNP@PDA2-10min at different holding times were calculated by refinement and quantitative analysis of the PXRD patterns, and the specific data are listed in Table S6. As illustrated in Figure 8a, the decomposition molar ratio of *γ*-CL-20 in CL-20/MTNP@PDA2-10min was lower than that of CL-20/MTNP cocrystal at the initial stage of heat treatment (<100 min), while it became higher than that of CL-20/MTNP cocrystal at the late stage of heat treatment (≥100 min). In the initial stage of heat treatment, PDA decomposed or melted, absorbing part of the heat to protect the cocrystal. However, at the late stage of heat treatment, the decomposition rate of CL-20/MTNP@PDA2-10min (97.6% of structure decomposed within 400 min) is higher than that of CL-20/MTNP cocrystal (92.8% of structure decomposed within 400 min), indicating that PDA coating cannot effectively inhibit the thermal decomposition of CL-20/MTNP cocrystal.

Through the TG-DSC tests of CL-20/MTNP cocrystal before and after coating, as shown in Figure 8b and Figure S16, it can be found that during the heating process, PDA exhibits a more significant endothermic effect and weight loss than CL-20/MTNP cocrystal, which may be the main reason why the weight loss of CL-20/MTNP@PDA composite is rather more severe than that of uncoated CL-20/MTNP cocrystal. In addition, PDA decomposes under thermal stimulation, which is speculated to be the key inducement for the significant acceleration of the decomposition rate of CL-20/MTNP@PDA after being held at 180 °C for 100 min in in-situ XRD tests. Meanwhile, it is found that the weight loss of CL-20/MTNP@PDA is higher than that of CL-20/MTNP cocrystal, which can be attributed to the fact that PDA can decompose at a relatively low temperature, leading to an increase in the weight loss rate of the coated explosive; at the same time, the heat released during the decomposition of PDA causes a local temperature rise, which further affects the initial decomposition temperature of the coated CL-20/MTNP cocrystal.

**Figure 9:**
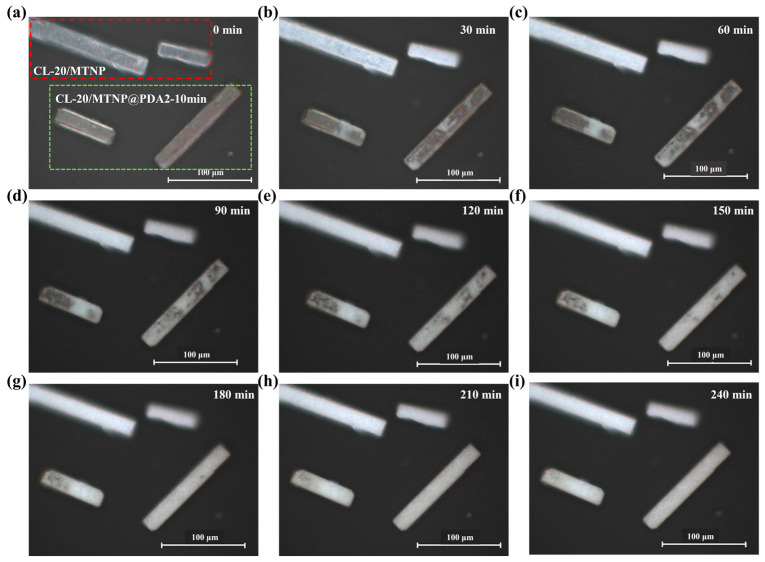
In-situ surface morphology evolution of CL-20/MTNP and CL-20/MTNP@PDA2-10min upon thermal treatment at 140 °C for various durations in air: (**a**) 0 min; (**b**) 30 min; (**c**) 60 min; (**d**) 90 min; (**e**) 120 min; (**f**) 150 min; (**g**) 180 min; (**h**) 210 min; (**i**) 240 min. Note: 140 °C is already above the decomposition temperature of CL-20. During the experiment, risk control measures need to be strictly implemented.

The morphological changes of CL-20/MTNP cocrystal and CL-20/MTNP@PDA2-10min at 140 °C were observed by in-situ hot-stage microscopy. As shown in Figure 9a–i, the surface of CL-20/MTNP crystals shows obvious whitening from 30 min, indicating the generation of white striped cracks and defects on the crystal surface. In contrast, the time required for the surface of CL-20/MTNP@PDA2-10min to change from brown to white is significantly longer than that of CL-20/MTNP cocrystal, and the PDA coated on the surface is not completely peeled off until 180 min. These results indicate that the critical threshold for crack and defect generation of CL-20/MTNP crystals after coating is increased under thermal stimulation, demonstrating that PDA coating at the initial stage can improve the thermal stability of CL-20/MTNP cocrystal. This conclusion is consistent with TG-DSC analysis that dopamine coating can absorb part of the heat at the initial stage and exert a certain thermal protection effect on CL-20/MTNP cocrystal. However, polydopamine decomposes at low temperatures, which not only weakens its thermal protection for the cocrystal but may even promote its thermal decomposition after polydopamine degradation.

## 4. Conclusions

The crystal structure stability of energetic cocrystals in chemical environments (water and aqueous mixed solvents) is critical for their safe preparation, processing, storage, and application. In contrast to most of the existing literature, which focuses primarily on the thermally stimulated decomposition of energetic cocrystals, this study quantitatively investigates the phase composition evolution and structural decomposition behavior of CL-20/MTNP energetic cocrystals in pure water and aqueous mixed solvent systems using PXRD-based phase refinement and quantitative analysis. CL-20/MTNP decomposes into *α*-CL-20 in both pure water and aqueous organic solutions, which is completely different from the *γ*-CL-20 phase formed under thermal stimulation. Furthermore, this study established a quantitative characterization method for cocrystal decomposition based on PXRD refinement. Using Jana2020 full-pattern fitting and Rietveld quantification, the accurate calculation of the volume fraction and molar fraction of the cocrystal phase and *α*-CL-20 phase during CL-20/MTNP decomposition has been achieved, and the quantitative dependence of decomposition extent on temperature, soaking time, and solvent type has been revealed. Compared with conventional qualitative PXRD analysis, this method provides comparable and repeatable stability evaluation data, offering a standardized approach for assessing the environmental stability of energetic cocrystals. Based on the channel-structured mechanism that facilitates MTNP escape from CL-20/MTNP, PDA coating is employed to encapsulate the cocrystal surface. This strategy physically blocks the MTNP diffusion channels and prevents solvent intrusion, thereby significantly suppressing structural decomposition in aqueous environments. It provides a new route for the modification of high-energy cocrystals in wet forming and humid storage scenarios.

This study focuses on the structural stability of CL-20/MTNP cocrystals in pure water and organic solvent/water environments. Future work could extend the investigation to other chemical environments, such as solutions with different pH values and solvent vapor atmospheres. Although rapid PDA coating has been successfully used to regulate the surface of CL-20/MTNP, yielding CL-20/MTNP@PDA2-10min with a smooth surface and remarkably improved stability in solvent environments, the underlying mechanism of this stabilization effect still requires further experimental verification and in-depth exploration. In addition, this work reveals decomposition laws from macroscopic phase changes, which have certain limitations. The molecular diffusion and interfacial evolution have not been directly observed. In the future, in-situ IR, in-situ Raman, in-situ electron microscopy, and other techniques could be integrated to monitor in real-time dynamic processes of water/solvent molecules entering the lattice, MTNP leaching, and CL-20 phase transition, so as to establish an atomic-scale decomposition kinetic model. Moreover, given the significant differences in the effects of various organic solvents on stability, theoretical calculations, such as binding energy, hydrogen bond strength, and diffusion energy barrier between solvents and the cocrystal surface, can be performed to reveal the nature of solvent effects and provide a predictive basis for screening highly stable solvent systems.

## Figures and Tables

**Table 1:** The average mole fraction of *α*-CL-20 from CL-20/MTNP cocrystal soaked in pure water at 40, 50, 60 and 70 °C for 8, 16, 24, and 48 h.

Soaking Time/h	Temperature/°C	*χ_α_*_-CL-20_/%
8	40	4.3 ± 0.4
	50	12.4 ± 0.7
	60	20.6 ± 0.8
	70	42.0 ± 4.3
16	40	6.6 ± 0.4
	50	14.3 ± 0.8
	60	23.1 ± 1.8
	70	50.9 ± 4.8
24	40	5.4 ± 1.4
	50	14.0 ± 0.3
	60	21.3 ± 0.9
	70	50.7 ± 2.3
48	40	6.8 ± 1.3
	50	16.7 ± 2.7
	60	27.6 ± 0.9
	70	66.2 ± 5.1

**Table 2:** The average mole fraction of *α*-CL-20 from CL-20/MTNP cocrystal soaked for 24 h in four different aqueous mixed solvents at 40, 50, 60, and 70 °C.

Solvent Type	Temperature/°C	*χ_α_*_-CL-20_/%
10 mmol/L AC	40	5.3 ± 0.6
	50	12.2 ± 1.7
	60	25.1 ± 0.7
	70	53.9 ± 5.1
10 mmol/L DMF	40	6.2 ± 0.2
	50	12.7 ± 0.5
	60	23.3 ± 0.5
	70	53.6 ± 1.7
10 mmol/L DMSO	40	5.5 ± 0.3
	50	11.1 ± 0.6
	60	25.8 ± 2.9
	70	49.2 ± 3.5
10 mmol/L EtOH	40	6.0 ± 0.2
	50	12.0 ± 1.2
	60	24.1 ± 3.1
	70	57.3 ± 4.0

## Data Availability

The original contributions presented in this study are included in the article/Supplementary Material. Further inquiries can be directed to the corresponding authors.

## Supplementary Materials

The following supporting information can be downloaded at: https://www.mdpi.com/article/10.3390/molecules31142527/s1. Figure S1: The PXRD refined results of raw CL-20/MTNP cocrystal and the CL-20/MTNP cocrystal after recrystallization; Figure S2: PXRD refined results of the CL-20/MTNP cocrystal at 70 °C for 48 h in a pure water environment with α-CL-20, γ-CL-20 and ε-CL-20 cif; Figure S3: Quantitative refined image of the decomposition products of the CL-20/MTNP cocrystal after immersion at 40 °C for different periods of time; Figure S4: Quantitative refined image of the decomposition products of the CL-20/MTNP cocrystal after immersion at 50 °C for different periods of time. Figure S5: Quantitative refined image of the decomposition products of the CL-20/MTNP cocrystal after immersion at 60 °C for different periods of time; Figure S6: Quantitative refined image of the decomposition products of the CL-20/MTNP cocrystal after immersion at 70 °C for different periods of time; Figure S7: The mole fraction of α-CL-20 in the decomposition products of the CL-20/MTNP cocrystal after being soaked in different temperatures in pure water environment; Figure S8: The refinement of the quantified decomposition products of the CL-20/MTNP cocrystal after being immersed in 10 mmol/L AC aqueous solution at different temperatures for 24 h; Figure S9: The refinement of the quantified decomposition products of the CL-20/MTNP cocrystal after being immersed in 10 mmol/L DMF aqueous solution at different temperatures for 24 h; Figure S10: The refinement of the quantified decomposition products of the CL-20/MTNP cocrystal after being immersed in 10 mmol/L DMSO aqueous solution at different temperatures for 24 h; Figure S11: The refinement of the quantified decomposition products of the CL-20/MTNP cocrystal after being immersed in 10 mmol/L EtOH aqueous solution at different temperatures for 24 h; Figure S12: The variation of mole fraction of α-CL-20 from CL-20/MTNP cocrystal soaked for 24 h at different temperatures in 10 mmol/L aqueous mixed solution; Figure S13: The PXRD of CL-20/MTNP before and after PDA coating; Figure S14: SEM images of CL-20/MTNP cocrystal, CL-20/MTNP@PDA1, CL-20/MTNP@PDA2-10min and CL-20/MTNP@PDA2-20min; Figure S15: Isothermal 180 °C in-situ XRD pattern of CL-20/MTNP and CL-20/MTNP@PDA2-10min.; Figure S16: The DSC curves of CL-20/MTNP, pure PDA, CL-20/MTNP@PDA2-10min, and CL-20/MTNP@PDA2-20min; Table S1: Crystallographic data and characteristic peaks of CL-20/MTNP, α-CL-20, γ-CL-20 and ε-CL-20; Table S2: The volume fraction of α-CL-20 in the product after soaking CL-20/MTNP cocrystal soaked in pure water at different temperatures.; Table S3: The mole fraction of α-CL-20 in the product after soaking CL-20/MTNP cocrystal soaked in pure water at different temperatures; Table S4: The volume fraction of α-CL-20 in the product after soaking CL-20/MTNP cocrystal in four different mixed solvents at different temperatures for 24 h; Table S5: The mole fraction of α-CL-20 in the product after soaking CL-20/MTNP cocrystal in four different mixed solvents at different temperatures for 24 h; Table S6: The mole fraction of γ-CL-20 in CL-20/MTNP and CL-20/MTNP@PDA2-10min at 180 °C during in-situ XRD with different holding times.
