# Supplementary material for: Crystal Structure Stability of CL-20/MTNP Energetic Cocrystals Without/with Polydopamine Coating in a Water/Organic Solvent Environment
Source: Molecules. 2026 Jul 20;31(14):2527. doi: 10.3390/molecules31142527 (PMC13415383; doi:10.3390/molecules31142527)
Supplement: Supplementary file 1 [file molecules-31-02527-s001.zip › molecules-4400705-supplementary.pdf]

Crystal Structure Stability of CL-20/MTNP Energetic Cocrystals without/with polydopamine coating in Water/Organic Solvent Environment

Peilin Yang [1,2], Yiru Chen [2], Chunbo Shi [2], Gang Li [2], Jinkun Guo [1,2], Kezhen Lv [2], Yeming Huang [2], Yijuan Liu [2], Yu Liu [2], Shiliang Huang [2,*] and Xiaoan Wei [1,*]

[1] School of Chemistry and Chemical Engineering, Nanjing University of Science and Technology, Nanjing 210094, P. R. China

[2] National Key Laboratory of Chemical Explosion Safety, Institute of Chemical Materials, China Academy of Engineering Physics, Mianyang, 621999, PR China

List of Contents

Detailed phase analysis of PXRD data

Figure S1. The PXRD refined results of raw CL-20/MTNP cocrystal and the CL-20/MTNP cocrystal after recrystallization.

Figure S2. PXRD refined results of the CL-20/MTNP cocrystal at 70 ℃ for 48 h in a pure water environment with α-CL-20, γ-CL-20 and ε-CL-20 cif.

Figure S3. Quantitative refined image of the decomposition products of the CL-20/MTNP cocrystal after immersion at 40 ℃ for different periods of time.

Figure S4. Quantitative refined image of the decomposition products of the CL-20/MTNP cocrystal after immersion at 50 ℃ for different periods of time.

Figure S5. Quantitative refined image of the decomposition products of the CL-20/MTNP cocrystal after immersion at 60 ℃ for different periods of time.

Figure S6. Quantitative refined image of the decomposition products of the CL-20/MTNP cocrystal after immersion at 70 ℃ for different periods of time.

Figure S7. The mole fraction of α-CL-20 in the decomposition products of the CL-20/MTNP cocrystal after being soaked in different temperatures in pure water environment.

Figure S8. The refinement of the quantified decomposition products of the CL-20/MTNP cocrystal after being immersed in 10 mmol/L AC aqueous solution at different temperatures for 24 h.

Figure S9. The refinement of the quantified decomposition products of the CL-20/MTNP cocrystal after being immersed in 10 mmol/L DMF aqueous solution at different temperatures for 24 h.

Figure S10. The refinement of the quantified decomposition products of the CL-20/MTNP cocrystal after being immersed in 10 mmol/L DMSO aqueous solution at different temperatures for 24 h.

Figure S11. The refinement of the quantified decomposition products of the CL-20/MTNP cocrystal after being immersed in 10 mmol/L EtOH aqueous solution at different temperatures for 24 h.

Figure S12. The variation of mole fraction of α-CL-20 from CL-20/MTNP cocrystal soaked for 24 h at different temperatures in 10 mmol/L aqueous mixed solution.

Figure S13. The PXRD of CL-20/MTNP before and after PDA coating.

Figure S14. SEM images of CL-20/MTNP cocrystal, CL-20/MTNP@PDA1, CL-20/MTNP@PDA2-10min and CL-20/MTNP@PDA2-20min.

Figure S15. Isothermal 180 ℃ in-situ XRD pattern of CL-20/MTNP and CL-20/MTNP@PDA2-10min.

Figure S16. The DSC curves of CL-20/MTNP, pure PDA, CL-20/MTNP@PDA2-10min, and CL-20/MTNP@PDA2-20min.

Table S1. Crystallographic data and characteristic peaks of CL-20/MTNP, α-CL-20, γ-CL-20 and ε-CL-20.

Table S2. The volume fraction of α-CL-20 in the product after soaking CL-20/MTNP cocrystal soaked in pure water at different temperatures.

Table S3. The mole fraction of α-CL-20 in the product after soaking CL-20/MTNP cocrystal soaked in pure water at different temperatures.

Table S4. The volume fraction of α-CL-20 in the product after soaking CL-20/MTNP cocrystal in four different mixed solvents at different temperatures for 24 h.

Table S5. The mole fraction of α-CL-20 in the product after soaking CL-20/MTNP cocrystal in four different mixed solvents at different temperatures for 24 h.

Table S6. The mole fraction of γ-CL-20 in CL-20/MTNP and CL-20/MTNP@PDA2-10min at 180 ^{o}C during in-situ XRD with different holding times.

Detailed phase analysis of PXRD data

Component identification: After the experimental PXRD data and crystal structure data (CIF) of the CL-20/MTNP cocrystal as well as possible crystal phases were imported into Jana2020, the Le Bail fitting (refinement without fixed atomic coordinate information) was performed to identify all crystalline phases from the collected PXRD patterns. During the Le Bail stage, zero-point shift, unit cell parameters, peak shape functions and background parameters were refined, while atomic positions were not involved.

Phase quantification: After confirming all existing crystalline phases and achieving full convergence of structural parameters, the output refinement file (m50 file) was transferred to conduct quantitative Rietveld refinement for phase volume fraction calculation, with atomic coordinates fixed throughout the quantitative stage. Specifically, the m50 file, a core proprietary project file format of the Jana2020 crystallographic refinement program, stored all complete experimental and structural input information. It could be opened by using Jana2020, and then the refinement mode would be changed from Le Bail mode to Rietveld mode, while retaining the original cell parameters, zero-point drift, and peak shape functions, and confirming that the atomic positions were fixed. Then, the overall polarity factor (T_{Overall}), an integrated scaling coefficient, correlates the calculated diffraction intensity from crystal structure models with the measured experimental PXRD intensity. During quantitative phase refinement, T_{Overall} optimized iteratively to match the total diffraction signal intensity of all crystalline phases. Combined with the refined volume fraction parameter phvol2 of secondary phases, the converged T_{Overall} value enables accurate calculation of the volume percentage of each individual crystal phase in the mixed solid residue.

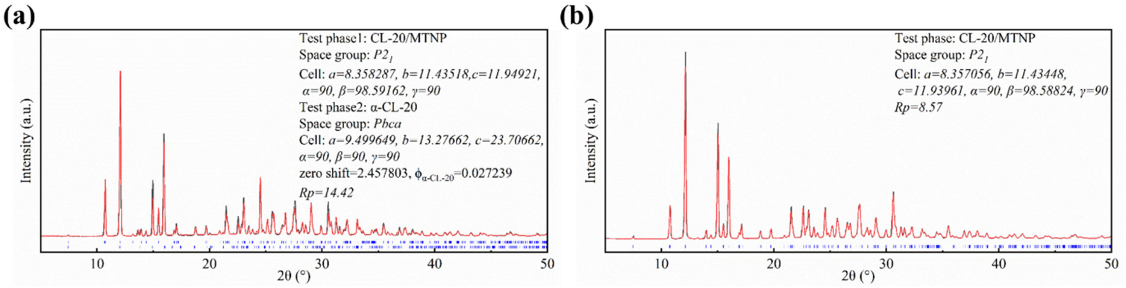

Figure S1. (a) The PXRD refined results of raw CL-20/MTNP cocrystal. (b) Final refinement of the CL-20/MTNP cocrystal after recrystallization.

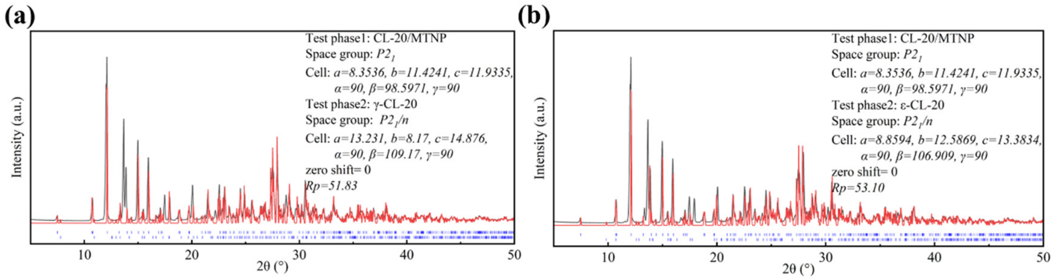

Figure S2. CL-20/MTNP cocrystal treated at 70 ℃ for 48 h in a pure water environment. (a) The PXRD refined results of the CL-20/MTNP cocrystal with γ-CL-20 cif. (b) The PXRD refined results of the CL-20/MTNP cocrystal with ε-CL-20 cif.

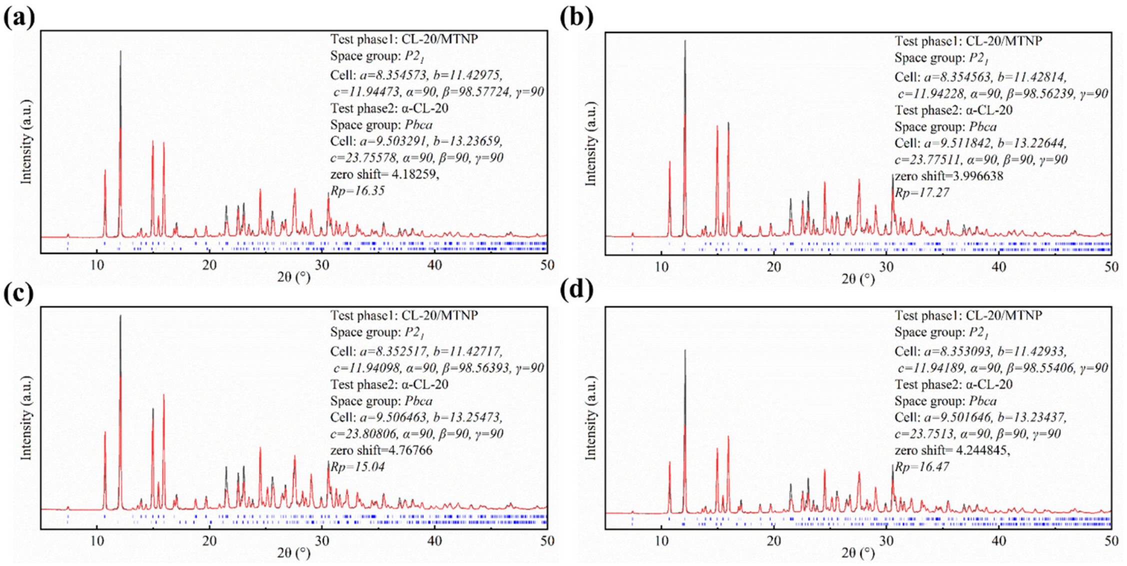

Figure S3. Quantitative refined image of the decomposition products of the CL-20/MTNP cocrystal after immersion at 40 ℃ for different periods of time: (a) 8 h, (b) 16 h, (c) 24 h, (d) 48 h. (The PXRD data are from the 1# experiment of the three parallel trials.)

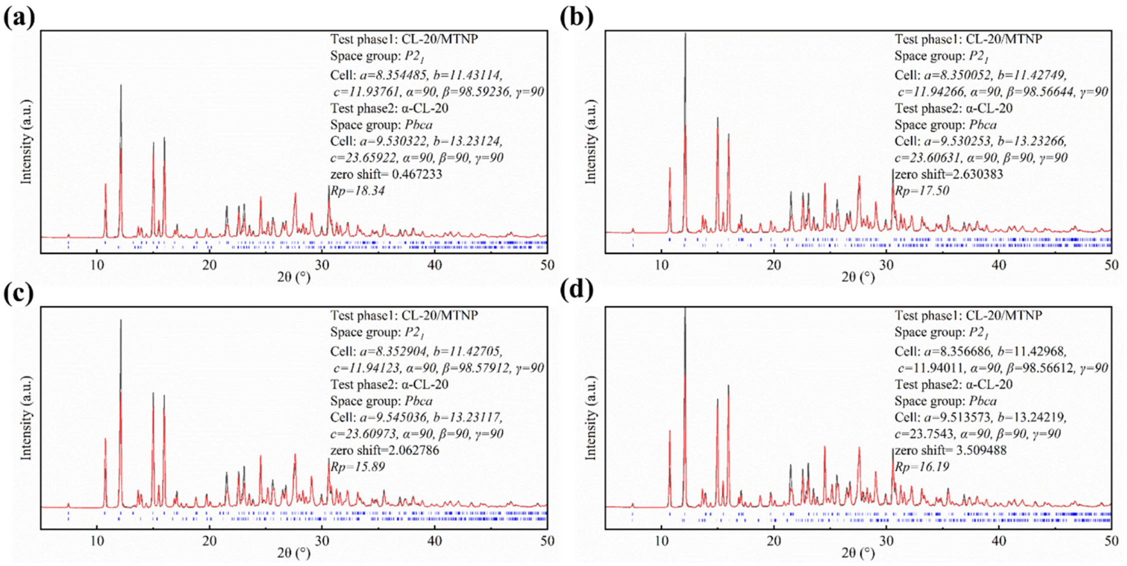

Figure S4. Quantitative refined image of the decomposition products of the CL-20/MTNP cocrystal after immersion at 50 ℃ for different periods of time: (a) 8 h, (b) 16 h, (c) 24 h, (d) 48 h.

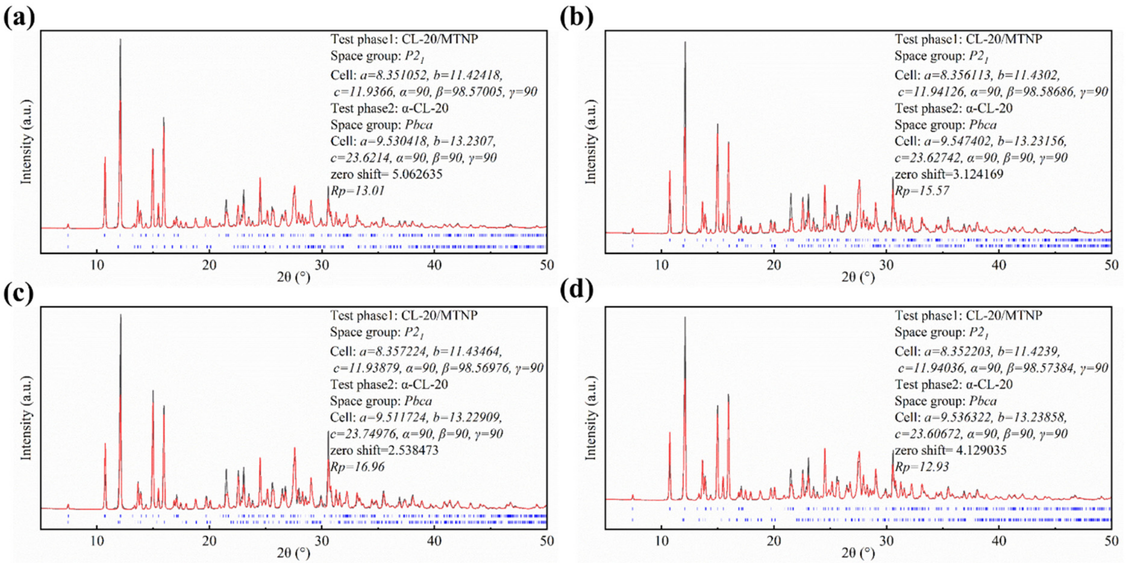

Figure S5. Quantitative refined image of the decomposition products of the CL-20/MTNP cocrystal after immersion at 60 ℃ for different periods of time: (a) 8 h, (b) 16 h, (c) 24 h, (d) 48 h.

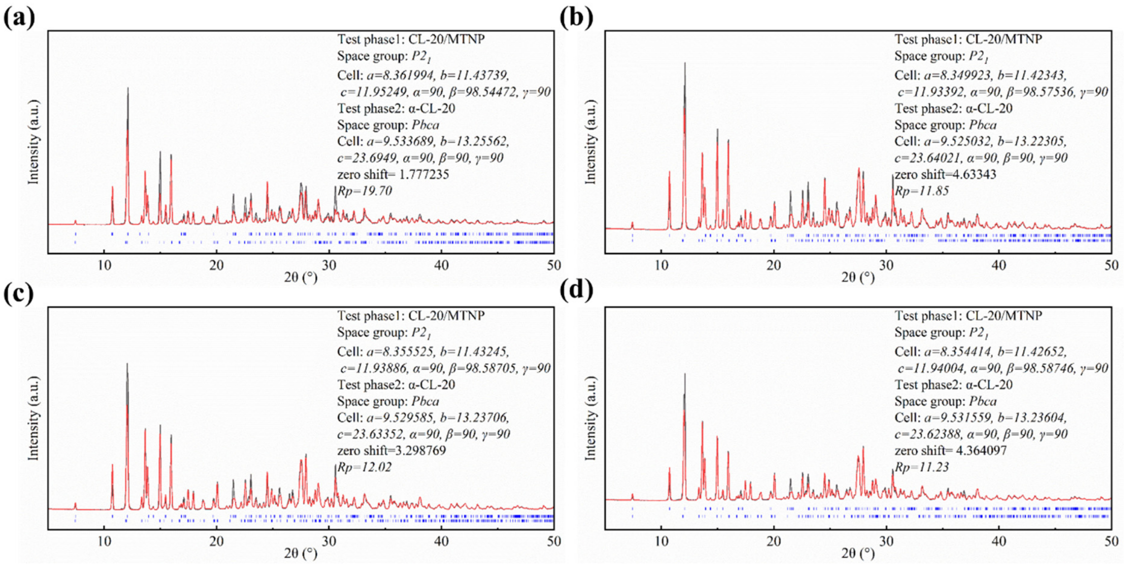

Figure S6. Quantitative refined image of the decomposition products of the CL-20/MTNP cocrystal after immersion at 70 ℃ for different periods of time: (a) 8 h, (b) 16 h, (c) 24 h, (d) 48 h.

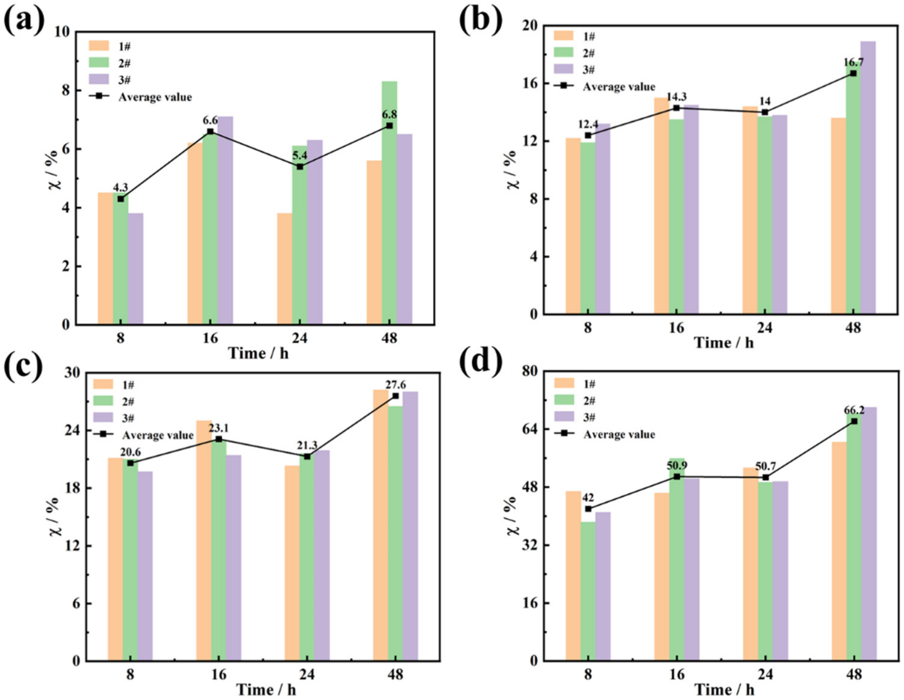

Figure S7. The mole fraction of α-CL-20 in the decomposition products of the CL-20/MTNP cocrystal after being soaked in different temperatures in pure water environment: (a) 40 ℃, (b) 50 ℃, (c) 60 ℃, (d) 70 ℃.

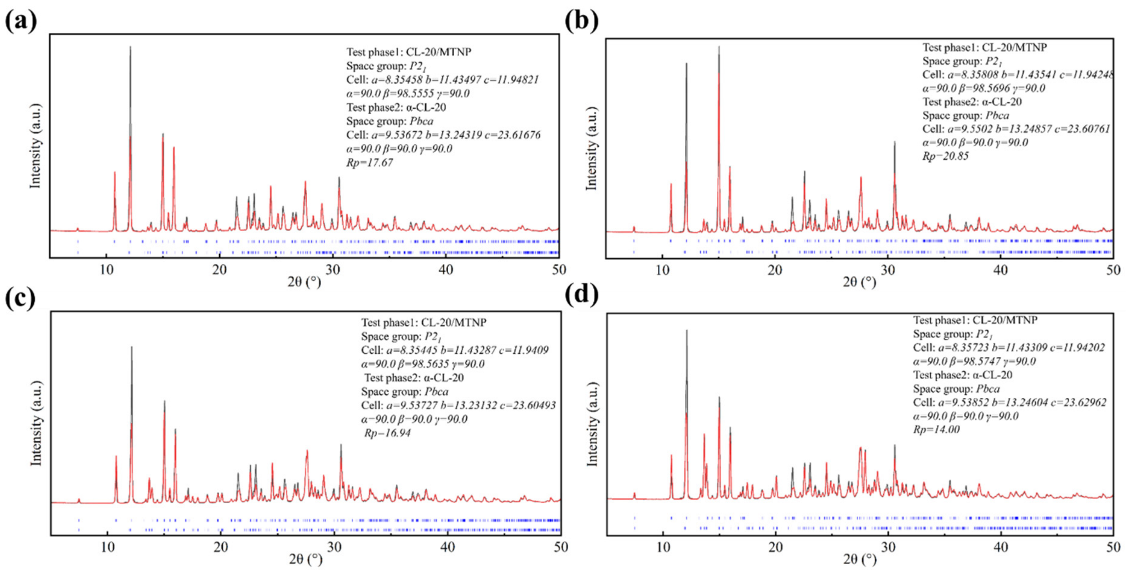

Figure S8. The refinement of the quantified decomposition products of the CL-20/MTNP cocrystal after being immersed in 10 mmol/L AC aqueous solution at different temperatures for 24 h: (a) 40 ℃, (b) 50 ℃, (c) 60 ℃, (d) 70 ℃.

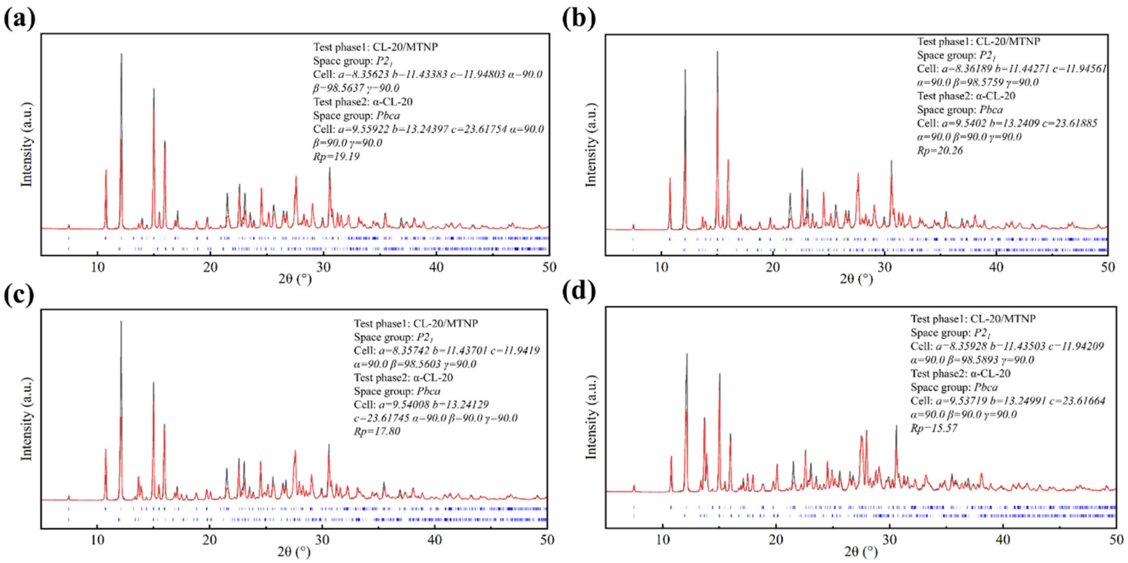

Figure S9. The refinement of the quantified decomposition products of the CL-20/MTNP cocrystal after being immersed in 10 mmol/L DMF aqueous solution at different temperatures for 24 h: (a) 40 ℃, (b) 50 ℃, (c) 60 ℃, (d) 70 ℃.

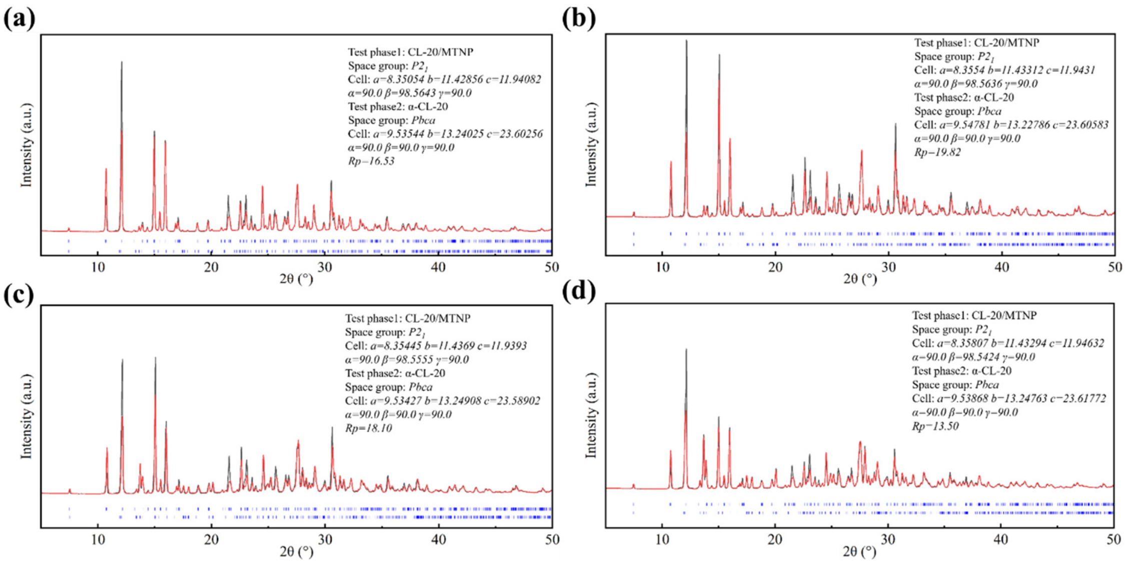

Figure S10. The refinement of the quantified decomposition products of the CL-20/MTNP cocrystal after being immersed in 10 mmol/L DMSO aqueous solution at different temperatures for 24 h: (a) 40 ℃, (b) 50 ℃, (c) 60 ℃, (d) 70 ℃.

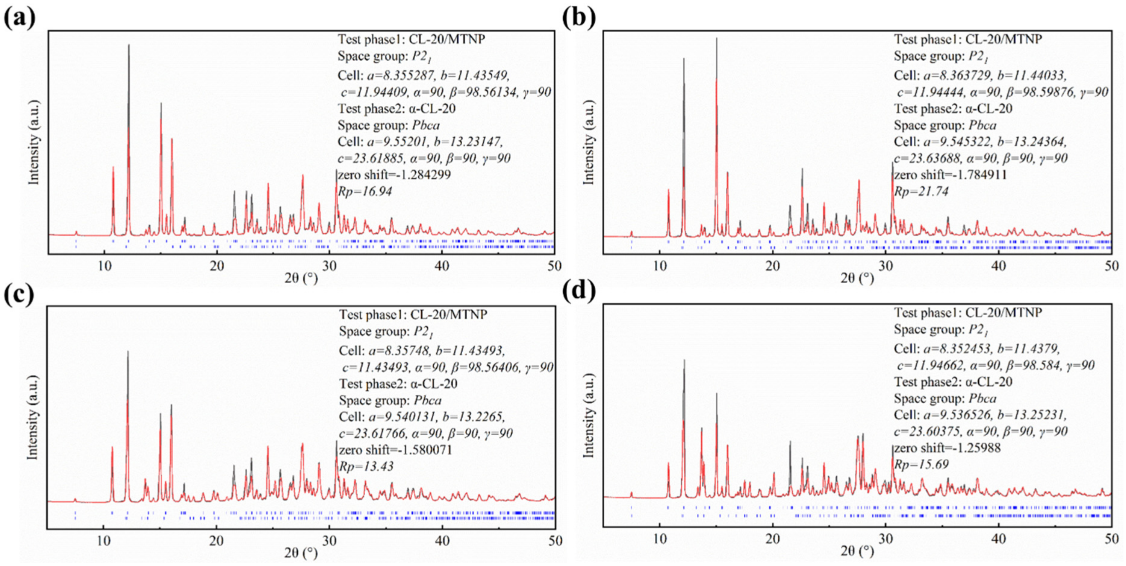

Figure S11. The refinement of the quantified decomposition products of the CL-20/MTNP cocrystal after being immersed in 10 mmol/L EtOH aqueous solution at different temperatures for 24 h: (a) 40 ℃, (b) 50 ℃, (c) 60 ℃, (d) 70 ℃.

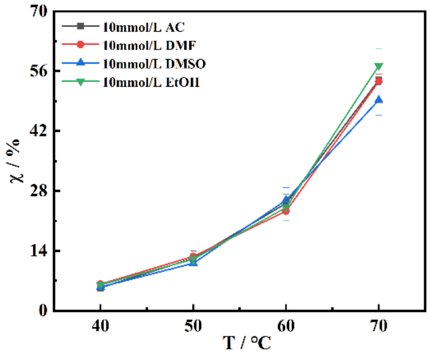

Figure S12. The variation of mole fraction of α-CL-20 from CL-20/MTNP cocrystal soaked for 24 h at 40, 50, 60 and 70 °C in 10 mmol/L aqueous mixed solution.

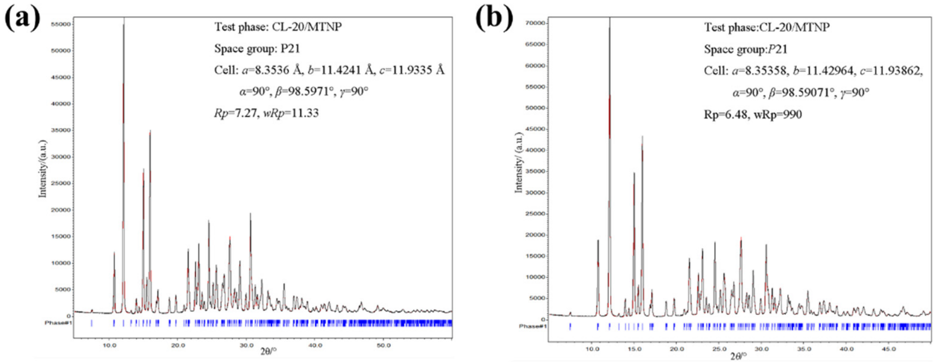

Figure S13. The PXRD of CL-20/MTNP (a) before and (b) after PDA coating.

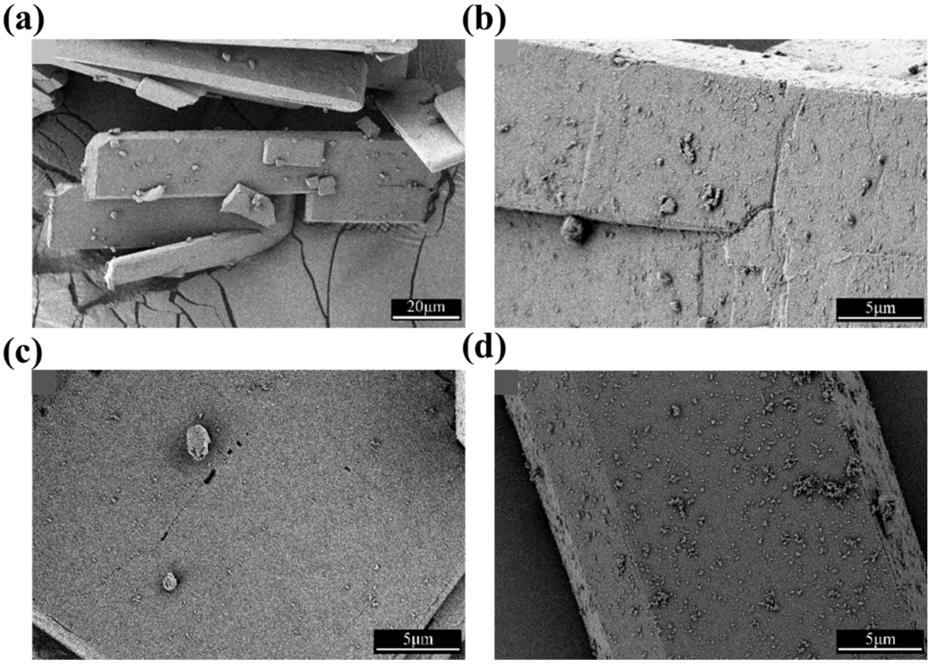

Figure S14. SEM images of (a) CL-20/MTNP cocrystal; (b) CL-20/MTNP@PDA1; (c) CL-20/MTNP@PDA2-10min; (d) CL-20/MTNP@PDA2-20min.

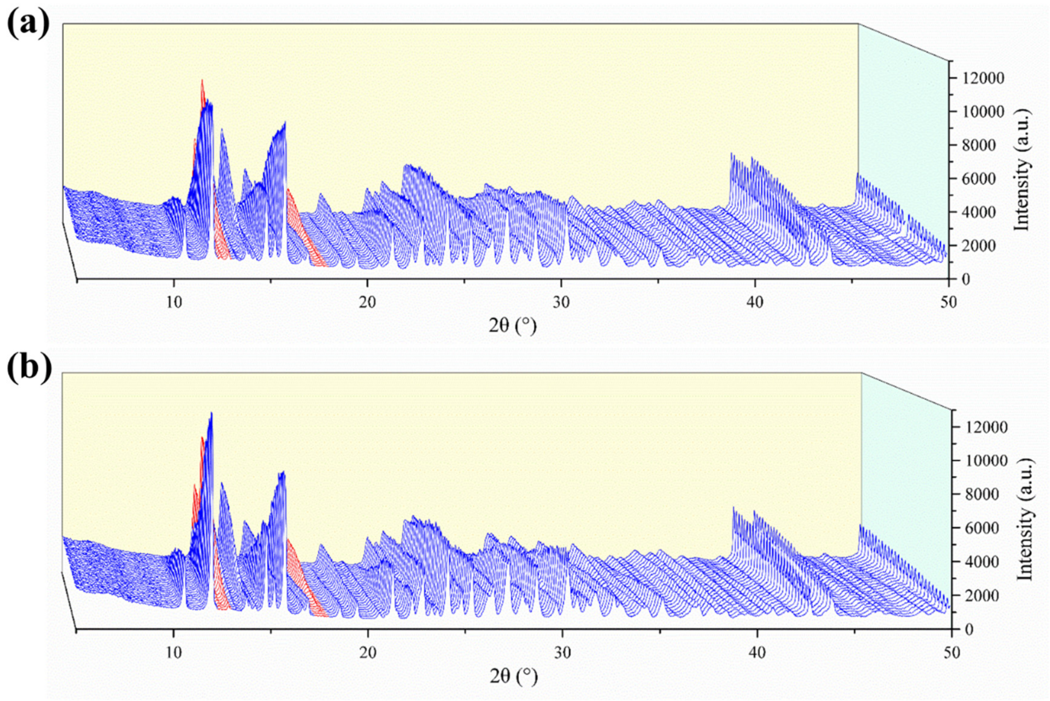

Figure S15. Isothermal 180 ℃ in-situ XRD pattern of (a) CL-20/MTNP; (b) CL-20/MTNP@PDA2-10min. (From front to back, the holding time at 180 ℃ is from 10 min to 400 min).

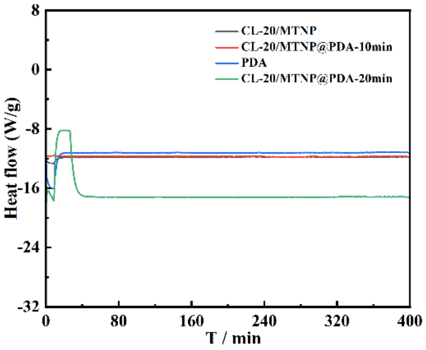

Figure S16. The DSC curves of CL-20/MTNP, pure PDA, CL-20/MTNP@PDA2-10min, and CL-20/MTNP@PDA2-20min.

Table S1. Crystallographic data and characteristic peaks of CL-20/MTNP, α-CL-20, γ-CL-20 and ε-CL-20.

Name	CL-20/MTNP	α-CL-20	γ-CL-20	ε-CL-20
Formula	C10H9N17O18	C6H6.5N12O12.25	C6H6N12O12	C6H6N12O12
Crystal System	monoclinic	orthorhombic	monoclinic	monoclinic
a/Å	8.35380(10)	9.546(1)	13.231(3)	8.852(2)
b/Å	11.42530(10)	13.232(1)	8.170(2)	12.556(3)
c/Å	11.94280(10)	23.634(2)	14.876(3)	13.386(3)
α/°	90	90	90	90
β/°	98.6370(10)	90	109.17(2)	106.82(2)
γ/°	90	90	90	90
Space Group	$P2_1$	*Pbca*	$P2_1/n$	$P2_1/n$
Characteristic peaks	10.70º, 12.11º, 14.99º, 15.98º, 24.60º	12.02º, 13.65º, 13.89º, 27.52º, 27.95º	12.55º, 12.93º, 13.33º, 14.16º, 25.26º	12.59º, 12.82º, 13.84º, 15.80º, 30.39º

Table S2. The volume fraction of α-CL-20 in the product after soaking CL-20/MTNP cocrystal in pure water at different temperatures.

Temperature / ℃	Soaking Time / h	Sample Number	Volume Fraction / %	Average Volume Fraction / %
40	8	1#	3.0	2.9±0.2
		2#	3.1	
		3#	2.6	
50		1#	4.3	4.5±0.2
		2#	4.5	
		3#	4.8	
60		1#	2.6	3.7±0.9
		2#	4.1	
		3#	4.3	
70		1#	3.8	4.6±0.9
		2#	5.7	
		3#	4.4	
40	16	1#	4.3	4.5±0.2
		2#	4.5	
		3#	4.8	
50		1#	10.6	10.1±0.5
		2#	9.5	
		3#	10.2	
60		1#	18.3	16.8±1.5
		2#	16.6	
		3#	15.4	
70		1#	36.6	41.0±4.7
		2#	45.9	
		3#	40.4	
40	24	1#	2.6	3.7±0.9
		2#	4.1	
		3#	4.3	
50		1#	10.1	9.8±0.2
		2#	9.6	
		3#	9.7	
60		1#	14.6	15.3±0.6
		2#	15.6	
		3#	15.8	
70		1#	43.3	40.8±2.2
		2#	39.4	
		3#	39.6	
40	48	1#	3.8	4.6±0.9
		2#	5.7	

	3#	4.4	
	1#	9.5	11.8±2.1
50	2#	12.4	
	3#	13.5	
	1#	20.8	20.3±0.7
60	2#	19.5	
	3#	20.6	
	1#	50.5	56.9±5.6
70	2#	59.1	
	3#	61.0	

Table S3. The mole fraction of α-CL-20 in the product after soaking CL-20/MTNP cocrystal in pure water at different temperatures.

Soaking Time / h	Temperature / ℃	Sample	$\chi_{\alpha\text{-CL-20}}$ / %
8	40	1#	4.5
		2#	4.5
		3#	3.8
	50	1#	12.2
		2#	11.9
		3#	13.2
	60	1#	21.1
		2#	21.0
		3#	19.7
	70	1#	46.8
		2#	38.3
		3#	41.0
16	40	1#	6.2
		2#	6.5
		3#	7.1
	50	1#	15.0
		2#	13.5
		3#	14.5
	60	1#	25.0
		2#	22.9
		3#	21.4
	70	1#	46.3
		2#	55.9
		3#	50.3
24	40	1#	3.8
		2#	6.1
		3#	6.3
	50	1#	14.4
		2#	13.7
		3#	13.8
	60	1#	20.3
		2#	21.6
		3#	21.9
	70	1#	53.3
		2#	49.3
		3#	49.5
48	40	1#	5.6
		2#	8.3

		3#	6.5
		1#	13.6
	50	2#	17.5
		3#	18.9
		1#	28.2
	60	2#	26.5
		3#	28.0
		1#	60.4
	70	2#	68.3
		3#	70.0

Table S4. The volume fraction of α-CL-20 in the product after soaking CL-20/MTNP cocrystal in four different mixed solvents at different temperatures for 24 h.

Solvent Type	Temperature / ℃	Sample	Volume Fraction / %
10 mmol/L AC	40	1#	3.4
		2#	3.4
		3#	4.1
	50	1#	9.7
		2#	7.2
		3#	8.8
	60	1#	18.3
		2#	18.9
		3#	17.7
	70	1#	38.4
		2#	48.1
		3#	45.5
10 mmol/L DMF	40	1#	4.1
		2#	4.2
		3#	4.4
	50	1#	9.3
		2#	8.6
		3#	8.7
	60	1#	16.6
		2#	17.3
		3#	16.7
	70	1#	44.1
		2#	45.0
		3#	41.8
10 mmol/L DMSO	40	1#	3.6
		2#	3.8

		3#	3.9
		1#	7.9
	50	2#	7.2
		3#	8.0
		1#	21.1
	60	2#	19.2
		3#	16.5
		1#	36.6
	70	2#	38.4
		3#	43.1
		1#	3.9
	40	2#	4.2
		3#	4.2
		1#	9.2
	50	2#	8.5
10 mmol/L EtOH		3#	7.5
		1#	15.4
	60	2#	20.2
		3#	17.1
		1#	43.8
	70	2#	51.9
		3#	46.4

Table S5. The mole fraction of α-CL-20 in the product after soaking CL-20/MTNP cocrystal in four different mixed solvents at different temperatures for 24 h.

Soaking Time / h	Temperature / ℃	Sample	$\chi_{\alpha\text{-CL-20}}$ / %
10 mmol/L AC	40	1#	4.9
		2#	5.0
		3#	6.0
	50	1#	13.8
		2#	10.3
		3#	12.6
	60	1#	25.1
		2#	25.8
		3#	24.4
	70	1#	48.2
		2#	58.0
		3#	55.5
10 mmol/L DMF	40	1#	6.0
		2#	6.2
		3#	6.4
	50	1#	13.2
		2#	12.3
		3#	12.5
	60	1#	22.9
		2#	23.8
		3#	23.1
	70	1#	54.1
		2#	55.0
		3#	51.7
10 mmol/L DMSO	40	1#	5.2
		2#	5.6
		3#	5.7
	50	1#	11.3
		2#	10.4
		3#	11.5
	60	1#	28.6
		2#	26.2
		3#	22.7
	70	1#	46.3
		2#	48.2
		3#	53.1
10 mmol/L DMF	40	1#	5.8
		2#	6.2
		3#	6.1
	50	1#	13.1

	2#	12.2
	3#	10.8
	1#	21.3
60	2#	27.5
	3#	23.6
	1#	53.8
70	2#	61.7
	3#	56.3

Table S6. The mole fraction of γ-CL-20 in CL-20/MTNP and CL-20/MTNP@PDA2-10min at 180 ℃ during in-situ XRD with different holding times.

Insulation Duration / min	Sample	$\chi_{\gamma\text{-CL-20}}$ / %
10	CL-20/MTNP	4.3
	CL-20/MTNP@PDA2-10min	4
55	CL-20/MTNP	6.4
	CL-20/MTNP@PDA2-10min	2.9
100	CL-20/MTNP	6.1
	CL-20/MTNP@PDA2-10min	6.8
130	CL-20/MTNP	9.7
	CL-20/MTNP@PDA2-10min	12.5
160	CL-20/MTNP	16.2
	CL-20/MTNP@PDA2-10min	23.2
205	CL-20/MTNP	28.3
	CL-20/MTNP@PDA2-10min	36.2
235	CL-20/MTNP	39.9
	CL-20/MTNP@PDA2-10min	57.2
280	CL-20/MTNP	58.8
	CL-20/MTNP@PDA2-10min	70.5
325	CL-20/MTNP	74.6
	CL-20/MTNP@PDA2-10min	70.8
400	CL-20/MTNP	92.8
	CL-20/MTNP@PDA2-10min	97.6
